# HIV-1 subtype C Envelope function becomes less sensitive to *N*-glycosylation deletion during disease progression

**DOI:** 10.1186/s13104-019-4375-0

**Published:** 2019-06-17

**Authors:** Evelyn Ngwa Lumngwena, Liliwe Shuping, Netanya Bernitz, Zenda Woodman

**Affiliations:** 1 Department of Medicine, Faculty of Health Sciences, University of Cape Town, Cape Town, South Africa; 2 Institute of Infectious Disease and Molecular Medicine, University of Cape Town, Cape Town, South Africa; 3 Centre for the Study of Emerging and Re-Emerging Infections (CREMER), Institute for Medical Research and Medicinal Plants Studies (IMPM), Ministry of Scientific Research and Innovation (MINRESI), Yaoundé, Cameroon; 4 Division of the National Laboratory Service, National Institute for Communicable Diseases, Johannesburg, South Africa; 5 Department of Biomedical Sciences, Faculty of Medicine and Health Sciences, Stellenbosch University, Stellenbosch, South Africa; 6 Department of Integrative Biomedical Sciences, Faculty of Health Sciences, University of Cape Town, Cape Town, South Africa

**Keywords:** HIV-1, Envelope *N*-glycosylation, Envelope function, Transmitted founder

## Abstract

**Objective:**

As part of a larger study to understand how Envelope *N*-glycosylation influences HIV-1 pathogenesis, we selected a participant infected with a single Subtype C variant and determined whether deletion of specific potential *N*-glycan sites (PNGs) impacted Envelope function longitudinally.

**Results:**

We deleted five PNGs previously linked to HIV-1 transmission of two matched Envelope clones representing variants at 5 and 173 weeks post-infection. The transmitted founder (TF) had significantly better pseudovirus entry efficiency than the chronic infection (CI) variant. Deletion of all PNGs significantly reduced TF entry efficiency, binding to dendritic cell-specific intracellular adhesion molecule 3 grabbing non-integrin (DC-SIGN) receptor and *trans*-infection. However, mutational analysis did not affect the phenotype of the CI Envelope to the same extent. Notably, deletion of the PNGs at N241 and N448 had no effect on CI Envelope function, suggesting that some PNGs might only be important during acute infection. Therefore, vaccines that elicit antibodies against *N*-glycans important for TF Envelope function could drive the loss of PNGs during immune escape, abrogating viral replication. Conversely, changes in *N*-glycosylation might have no effect on some variants, reducing vaccine efficacy. This finding highlights the need for further investigation into the role of Envelope *N*-glycosylation in HIV-1 pathogenesis.

## Introduction

Previous reports suggested that Envelope (Env) *N*-glycosylation is essential for HIV-1 transmission [1–4] and *N*-glycosylation patterns differ between transmitted founder (TF) and chronic infection (CI) variants [5]. Several different mechanisms have been suggested to explain the influence of Env *N*-glycosylation on the transmission of R5 tropic variants such as sensitivity to immune responses and enhanced binding to dendritic cell-specific intracellular adhesion molecule 3 grabbing non-integrin (DC-SIGN) and alpha4beta7 receptors via Env *N*-glycans [6–8]. Go et al. [5] found that TF Env was more heavily mannosylated than CI clones, markedly at conserved PNG sites N241, N262, N386, N392 and N448, suggesting that these sites might play a different role in Env function during acute infection than during the chronic stage. As part of a larger study focussed on the role of Env *N*-glycosylation in HIV-1 subtype C pathogenesis, we determined whether the loss of these PNGs impacted Env function differently over the course of infection.

As dendritic cells (DCs) are found within the female genital tract (FGT), express DC-SIGN that bind gp120 high mannose *N*-glycans and facilitate *trans*-infection of CD4 T cells in the lymph nodes [9, 10], we tested whether matched TF and CI variants from a subtype C single variant transmitted participant differed in mannosylation, binding to DC-SIGN and *trans*-infection. We hypothesised that if TF Envs were enriched with high mannose compared to CI variants then they would bind DC-SIGN with high affinity resulting in enhanced CD4^+^ T cell *trans*-infection [11]. When there was no clear relationship between Env *N*-glycosylation and DC-SIGN binding, we deleted PNGs at N241, N262, N386, N392 and N448 and determined Env function as these sites not only carried high mannose residues but N386 and N392 were suggested to be involved in binding DC-SIGN [12, 13].

## Main text

### Methods

#### Samples

Two full-length *envelopes* (*env*) were cloned from an HIV positive woman sampled at 5 and 173 weeks post-infection (wpi) from a larger cohort described in Abrahams et al. [14]. The original study obtained ethical approval from institutional review boards, and all participants provided written informed consent [5]. The two functional Env clones (TF and CI) were generated from single-genome-amplification (SGA)—derived PCR products and cloned into pcDNA/His-Topo (Invitrogen). [Accession numbers: FJ443350.1 and HQ625601 (https://www.ncbi.nlm.nih.gov)]. Sequences were aligned in Bio-edit.

#### Cell culture

Human embryonic kidney (HEK) 293 T cells (Gift from Carolyn Williamson, Institute of Infectious Diseases and Molecular Medicine, University of Cape Town & NHLS) and TZM-bl cells [NIH AIDS Reagent Program (ARP), Division of AIDS, NIAID from Dr. John C. Kappes, Dr. Xiaoyun Wu and Tranzyme Inc] were maintained in Dulbecco modified Eagle high glucose medium (DMEM) (Lonza, Whitehead Scientific) supplemented with 10% fetal bovine serum (FBS) (PAA, Biocom Biotech), 1 U/mL penicillin and 1 µg/mL streptomycin (Lonza, Whitehead Scientific). Raji cells [NIH-ARP from Alexandre Kabamba at the National Institute of Communicable disease (NICD), South Africa] and Raji DC-SIGN cells (NIH-ARP, from Drs. Li Wu and Vineet N. KewalRamani) were maintained in RPMI supplemented with 10% FBS. All cells were grown in a humidified incubator at 37 °C with 5% CO_2._

#### Generation of mutants

Modified Quikchange® site directed mutagenesis (Stratagene) was used to delete PNGs and insert stop codons in gp160. Complimentary primers carrying the desired mutations and silent restriction enzymes sites were used to amplify *env* with the following cycling conditions: 94 °C for 3 min followed by 20 cycles of 94 °C for 30 s, 55 °C or 58 °C for 30 s, 72 °C for 12 min and a final annealing step at 72 °C for 20 min using Phusion Hotstart (Thermo Scientific, USA). SDM PCR products were digested with *Dpn*I (Thermo Scientific®, USA) before competent *E.coli* (Promega) were transformed. Plasmid was extracted using the PureYield™ Plasmid Miniprep System (Promega) according to the manufacturer’s instructions. Mutations were confirmed by sequencing (CAF Stellenbosch, SA).

#### *N*-glycosylation analysis of Env

To express gp140, 4 × 10^5^ HEK 293 T cells were transfected with 6 µg of each *env* clone using PEI (Polyethyleneimine) (Sigma-Aldrich, USA). After transfection, HEK 293 T cell culture medium was diluted 1:1 with 1 mL of binding buffer (20 mM morpholineethanesulfonic acid, 130 mM NaCl, 10 mM CaCl_2_) at 4  C before the addition of 20 µL *Galanthus nivalis* agarose beads (Sigma-Aldrich®). Binding was carried out overnight at 4  C and the beads were washed twice with PBS (Whitehead Scientific) and then treated with either endo-β-*N*-acetylglucosaminidase H (EndoH) (0.5 U) or peptide *N*-glycosidase F (PNGaseF) (0.5 U) according to the manufacturers recommendations (New England Biolabs®) and then analysed by SDS-PAGE and Western blotting. Differences in *N*-glycosylation were determined by comparing the molecular weight (MW) of *N*-glycosylated (untreated), demannosylated (EndoH-treated) and fully deglycosylated gp140 (PNGaseF-treated) and the level of high mannose *N*-glycans was calculated as a percentage of total *N*-glycans using the formula: [(Untreated − EndoH-treated)/(Untreated) − (PNGaseF-treated)] × 100.

#### Pseudovirus production

HEK 293 T cells were transfected with 2.5 µg of gp160 and 5 µg of the viral backbone (pSG3ΔEnv). Culture medium was collected 48 h following transfection and filtered through a 0.22 µm pore size filter, FBS was adjusted to 20% and PSV was stored in single-use aliquots at − 70 °C.

#### p24 ELISA

A chemiluminescent p24 ELISA (Aalto Bio-reagents) and the TROPIX^®^ detection system (CDP-Star^®^, Applied Biosystems) was used to determine the concentration of PSV. Relative light units (RLU) were converted to p24 concentration (ng/mL) using a standard curve and non-linear regression analysis.

#### Pseudovirus entry assay

TZM-bl cells were seeded at 10^4^ cells per well before infection with fivefold serial dilutions of PSV for 48 h at 37 °C. All PSV were normalised to 100 ng/mL p24 prior to serial dilution and infectivity was measured by luminescence using BriteGlo® (Promega) and luminometer (Turner Biosystems®). Negative controls included background luminescence of wells carrying cells only or cells transfected with pSG3ΔEnv only.

#### DC-SIGN binding assay

Ten ng/mL p24 pseudovirus was added to Raji and Raji DC-SIGN cells at a density of 10^5^ cells/well in a total volume of 200 µL. After binding for 2.5 h at 37 °C, cells were washed four times by centrifugation (2500 rpm) with RPMI to remove all unbound virus, then lysed in 1% Empigen-TBS before determining cell-associated p24 (Aalto Biosystems).

#### Generation of monocyte derived dendritic cells (MDDCs)

PBMCs were obtained from healthy blood donor buffy coats by density gradient centrifugation using Ficoll-Hypaque (Sigma-Aldrich). Monocytes were isolated from PBMCs by positive selection using CD14+ coated beads (130-050-201 Miltenyi, USA or Biochom Biotech, SA) according to manufacturer’s instructions or by adherence. Monocytes were adhered in serum-free medium for two hours at 37 °C, 5% CO_2,_ non-adherent cells washed and differentiated in medium with 1000 U/mL GM-CSF (PHC2013, Biosource)*,* and 500 U/mL of recombinant human IL-4 (PHC0045, Biosource***)*** for 6 days with recombinant cytokines supplemented every other day.

#### *Trans*-infection

PSV was normalised using p24 ELISA and equal quantities of virus was used to infect TZM-bl cells directly and indirectly via binding first to Raji DC-SIGN cells and MDDCs. PSV was bound to 10^5^ Raji DC-SIGN cells or MDDCs and unbound virus was removed by washing with RPMI before adding to TZM-bl cells seeded at a density of 10^4^ cells/well. Cells were incubated at 37 °C with 5% CO_2_ for 48 h and pseudovirus entry was measured using BriteGlo® (Promega) and luminometer (Turner Biosystems® Modulus Microplate).

### Results and discussion

**Fig. 1:**
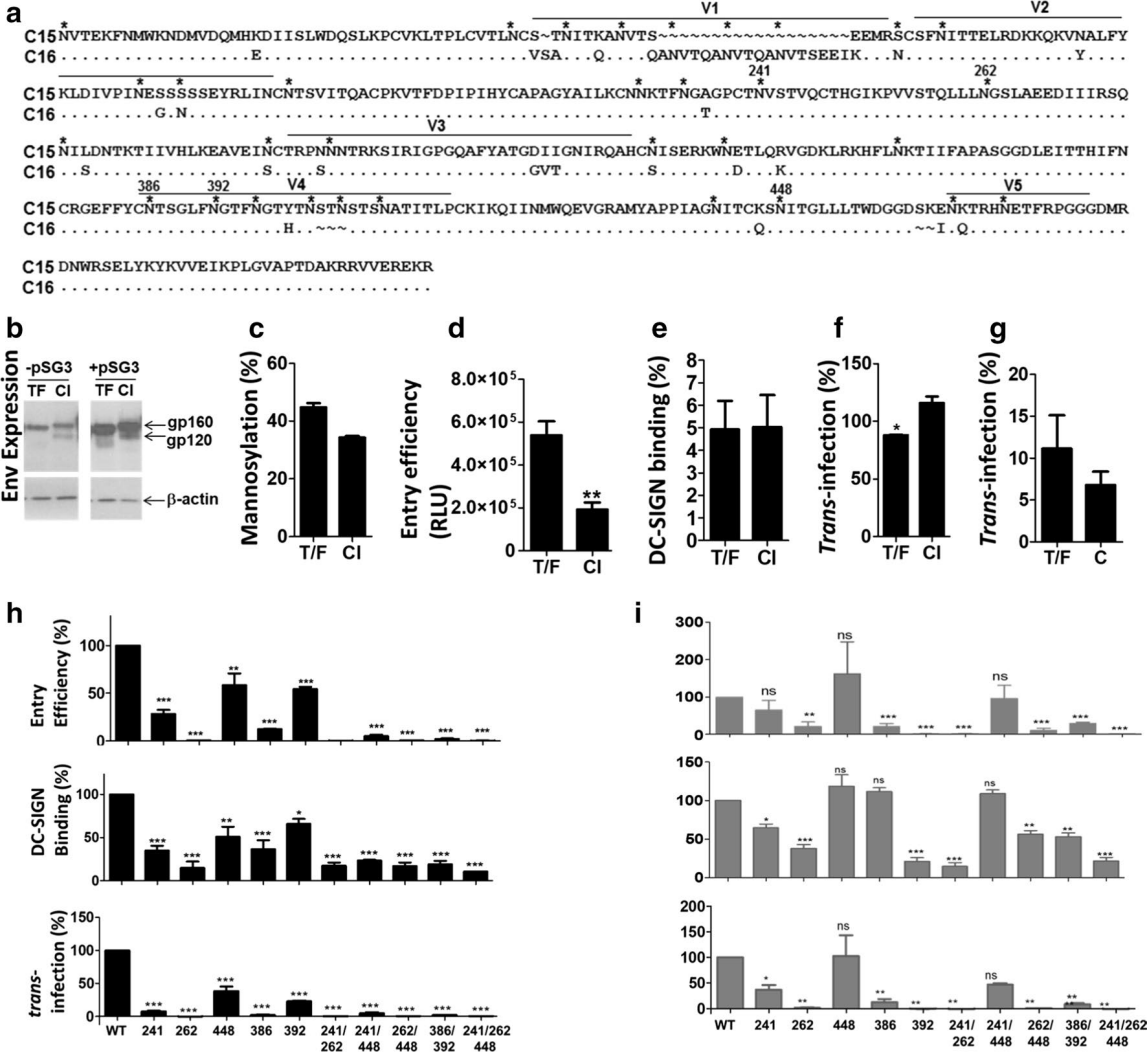
Phenotypic comparison of transmitted founder and matched chronic infection Envelopes. Env sampled from a participant at 5 (transmitted founder; TF) and 173 (chronic infection; CI) weeks post-infection were expressed in HEK293T cells. **a** The sequences of the two clones (C15 and C16) were aligned in Bioedit and PNGs are indicated with asterisk. Gp120 sequence is shown starting from the first potential N-glycan site (PNG) at position 88 (HXB2 numbering) with variable loops indicated. **b** Env expression was determined in the presence and absence of pSG3Δenv by Western blotting. β-actin, a protein loading control, gp160 and gp120 are indicated with arrows. A representative of three independent experiments is shown. **c** Gp140 was digested with EndoH and PNGaseF and the molecular weight (MW) was compared to that of undigested gp140 using Western blotting. Mannosylation (%) was determined using the equation: [(Untreated – EndoH-treated)/(Untreated) − (PNGaseF-treated)] × 100. The average of two biological repeats with standard error of the mean (SEM) are indicated. Statistical analysis was done using One-way Anova. Pseudovirus (PSV) was compared for their ability to **d** infect TZM-bl cells, **e** bind to Raji-DC-SIGN cells and *trans*-infect TZM-bl cells after capture by **f** Raji-DC-SIGN or **g** MDDCs. The average relative light units (RLU) of four independent entry efficiency experiments are indicated. DC-SIGN binding of three independent experiments was calculated relative to PSV input (%) and *trans*-infection of four biological experiments are indicated relative to the entry efficiency of equivalent PSV (%). Error bars represent SEM and statistical analysis was done using Mann Whitney U test. **h** TF and **i** CI Env potential N-glycan sites (PNGs) at N241, N262, N448, N386 and N392 (HXB2 numbering) were deleted by site-directed mutagenesis and compared to WT for their ability to infect TZM-bl cells, bind to DC-SIGN, and *trans*-infect TZM-bl cells. Bars indicate the mean of two independent experiments with SEM. Statistical analysis was done using One-way Anova and Bonferroni’s post-test. PSVs generated using pSG3∆Env and empty vector was used as a negative control (Ctrl). For all statistical analysis, *, ** and *** indicate p values < 0.05, p < 0.01 and p < 0.001, respectively relative to WT, ns was not significant

A CAPRISA 002 study participant, CAP239, was shown to be infected with a single variant at transmission through sequencing twenty SGA *env* PCR products at 5 wpi [14]. Two Envs were cloned at 5 and 173 wpi and selected for mutational analysis as they both carried N241, N262, N386, N392 and N448, unlike other clones (Fig. 1a), and these were compared for Env expression and mannosylation as well as PSV entry efficiency, DC-SIGN binding and *trans*-infection of CD4+ cells.

We first confirmed the expression of Env in the absence and presence of the PSV backbone, pSG3Δenv. Although expression increased with the co-transfection of pSG3Δenv, there was no difference between TF and CI Env expression (Fig. 1b). The CI clone had a higher MW than the TF and showed a clear gp120 band that was not apparent for the TF. However, when Env was digested with PNGaseF, a band corresponding to deglycosylated gp120 was observed (data not shown), indicating that TF Env was cleaved, albeit less efficiently than the CI clone. The higher apparent MW of CI Env is likely due to *N*-glycosylation as the predicted MW of the two clones differed by only 1 KDa. Although the Envs shared 20 conserved PNGS, seven PNGS were gained and five PNGS were lost so that the CI Env carried two additional PNGs (Fig. 1a). Envs were mutated to delete the transmembrane domain of gp41 to generate soluble gp140 constructs. When soluble gp140 was digested with EndoH and PNGaseF which demannosylate and deglycosylate, respectively, shifts in MW indicated that gp140 of the TF was more heavily mannosylated (45% vs 34%), suggesting that the CI clone carried more complex-type *N*-glycans (Fig. 1c). It is possible that the number of complex *N*-glycans, with negatively charged sialic acid moieties, led to more extensive smearing of CI gp160 during SDS PAGE, distorting its MW.

Changes in *N*-glycosylation have been shown to influence Env function [8]. When PSV was used to infect TZM-bl cells, the TF Env had threefold higher entry efficiency than the matched CI variant (Fig. 1d). This finding was supported by an earlier study that showed that infectious molecular clones (IMCs) carrying TF Envs from subtype C and subtype B HIV had twofold higher replication capacity than unmatched CI Envs [11]. Next, PSV, with known entry efficiency, was bound to either Raji DC-SIGN or monocyte derived dendritic cells (MDDCs) before adding to TZM-bl cells. The CI PSV was transferred significantly better to TZM-bl cells than TF by Raji-DC-SIGN cells (Fig. 1f). On the contrary, MDDCs apparently transferred TF PSVs better than CI virus (Fig. 1g). There was no difference in DC-SIGN binding between TF and CI PSVs (Fig. 1e), suggesting that MDDC-mediated *trans*-infection might be influenced by receptors other than DC-SIGN. Therefore, the only consistent significant difference between TF and CI Env phenotype was the ability to mediate entry of CD4+ TZM-bl cells.

The PNGs at positions 241, 262, 448, 386, and 392 were conserved across TF and CI Env sequences. To determine whether loss of mannose *N*-glycans and/or disruption of the potential DC-SIGN binding site affected Env function, the PNGs were deleted singly and in combination. Our data supports previous findings that showed N262 was important for Env function [15, 16]. However, PNG deletion affected the function of the Env clones differently: N392Q abrogated the function of CI Env but only reduced the entry efficiency of the TF clone, albeit significantly. Overall, TF Env was highly sensitive to *N*-glycan deletion as PSV entry efficiency, DC-SIGN binding and *trans*-infection of all mutants were significantly reduced compared to wild-type (WT) (Fig. 1h). On the contrary, CI Env was not affected to the same extent as deletion of PNGs at N241 and N448 had no significant effect on its function (Fig. 1i). Deletion of N448 seemed to increase the entry efficiency of C16 supporting a previous report that suggested loss of the *N*-glycan at this position affected the structure of the C4 region although this effect was not apparent for C15 [17]. Therefore, the overall *N*-glycan arrangement of CI Env might be able to compensate for the fitness cost associated with the loss of N241 and N448. On the contrary, the structure of TF Env seemed less able to accommodate the loss of any of the five PNGs, suggesting that these sites are important for Env entry efficiency during acute infection. When we compared the sequences of 589 Subtype C Env sequences (https://www.lanl.com), all five PNGs were conserved, supporting the suggestion that these sites play a very important role in Env function. However, it seems as though Env structure and function become less reliant on these PNGs during disease progression probably to accommodate immune escape mutations via the shifting glycan shield. C15 and C16 differed by 43 amino acids with the most marked difference within V1 that resulted in the introduction of 3 PNGs (Fig. 1a). Despite the five PNGs becoming less important for Env function over time, they are highly conserved across variants from chronic infection, suggesting that they might be important for alternative processes. Overall, the data suggests that the role of *N*-glycans in the maintenance of Env structure and function varies according to time post infection. This begs the question: how quickly do some *N*-glycans become obsolete? This has important ramifications for the design of vaccines that include *N*-glycan epitopes, as the loss or gain of PNGs associated with escape from neutralizing immune responses might drive changes in viral fitness that differ according to the HIV infected participant.

## Limitations

The limitation of the study is the small sample size.

## Data Availability

The data sets used and/or analyzed during the current study are available from the corresponding author without limitation (Accession numbers of samples: FJ443350.1 and HQ625601).

## Abbreviations

DC: dendritic cells
MDDC: monocyte derived dendritic cells
DC-SIGN: dendritic cell-specific intracellular adhesion molecule 3 grabbing non-integrin
FGT: female genital tract
PSV: pseudovirus
